# A New Linear Oscillatory Actuator with Variable Characteristics Using Two Sets of Coils

**DOI:** 10.3390/s16030377

**Published:** 2016-03-15

**Authors:** Fumiya Kitayama, Katsuhiro Hirata, Noboru Niguchi, Masashi Kobayashi

**Affiliations:** Department of Adaptive Machine Systems, Graduate School of Engineering, Osaka University, Osaka, 565-0871, Japan; k-hirata@ams.eng.osaka-u.ac.jp (K.H.); noboru.niguchi@ams.eng.osaka-u.ac.jp (N.N.); masashi.kobayashi@ams.eng.osaka-u.ac.jp (M.K.)

**Keywords:** linear oscillatory actuator, variable characteristics, finite element analysis

## Abstract

Nowadays, electromagnetic linear oscillatory actuators are used as vibration control devices because of their high controllability. However, there is a problem that thrust and vibration are small at a wide drive frequency range. In order to improve this problem, we propose a new linear oscillatory actuator that can easily change its own characteristics by using two sets of coils. Through finite element analysis, large vibration was observed at 100 Hz in a series connection, and large vibration and high thrust were observed at 70 Hz and 140 Hz in a parallel connection. From these results, we verified that the actuator had two different characteristics due to switchable connections, and could generate high thrust and large vibration by smaller currents at a wide drive frequency range.

## 1. Introduction

**Figure 1:**
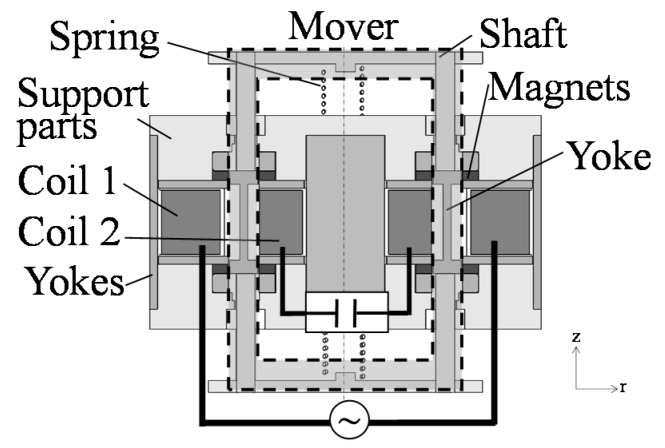
Basic construction of our previous actuator.

**Figure 2:**
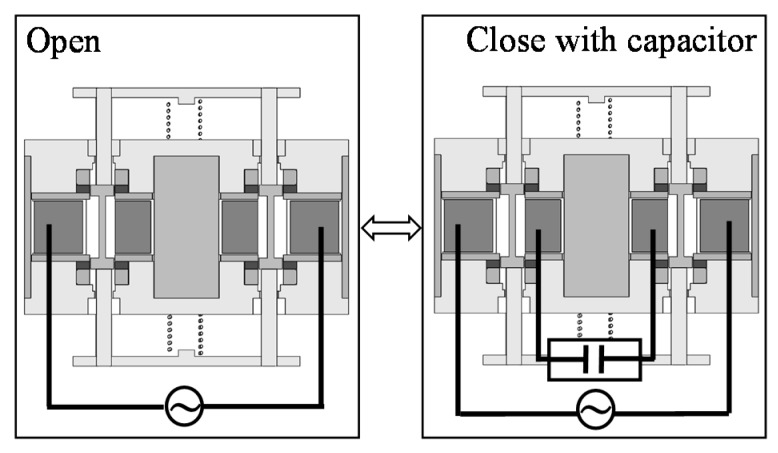
Connections of coils in previous actuator.

Recently, problems have been caused by mechanical vibrations [1,2]. Passengers often feel uncomfortable, especially in automobiles by a frame vibration that is generated by an engine. In order to solve the problem, active vibration control devices have been developed [3,4,5,6,7]. In these devices, many electromagnetic linear oscillatory actuators are used because of their high controllability. However, the electric power of these actuators is relatively high. Therefore, a high thrust and large vibration due to small currents have been required at a wide drive frequency range. In previous studies, actuators with improved magnetic circuits have been proposed [8,9]. However, the effectiveness of the improved magnetic circuit is still low for the high thrust and vibration characteristics. Here, we have developed an actuator using a regenerative energy, as shown in Figure 1 [10]. Furthermore, the actuator is verified to realize variable characteristics under high thrust and large vibration by changing the coil connection, as shown in Figure 2. However, the previous actuator has difficulty manufacturing because a mover is sandwiched by two stators. In this paper, a new and simple actuator that has variable characteristics for high thrust and large vibration is proposed [11]. The operational principle is described, and its characteristics are computed using finite element analysis. Finally, the effectiveness of the proposed actuator is discussed.

## 2. Proposed Actuator

**Figure 3:**
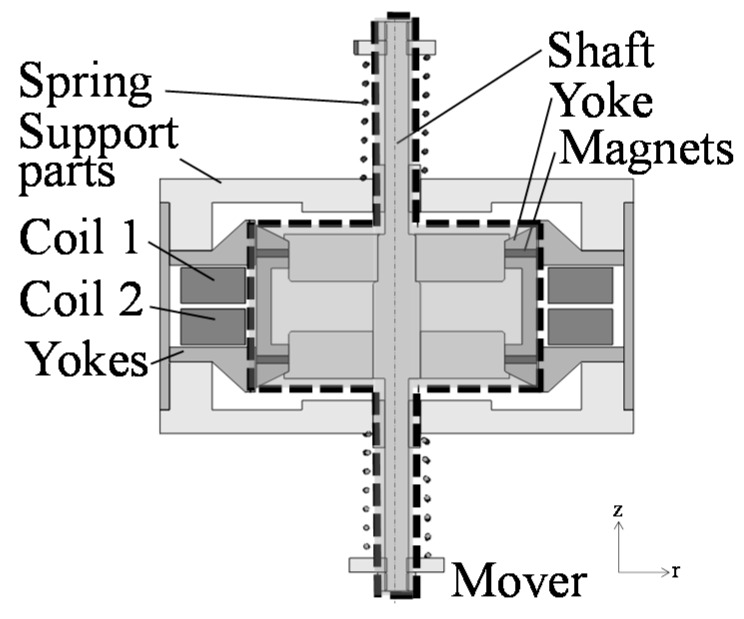
Basic construction of proposed actuator.

**Figure 4:**
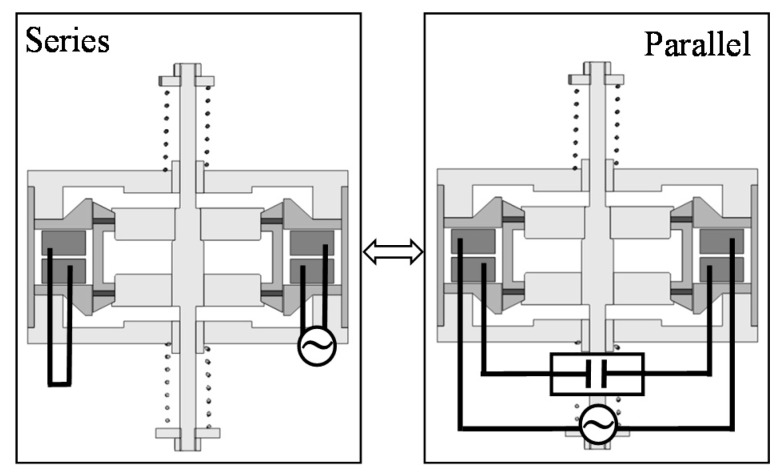
Connections of coils in proposed actuator.

The cross-section view and coil connection of the proposed actuator are shown in Figure 3 and Figure 4. In the proposed actuator, variable characteristics can be realized by changing the coil connection in series or parallel. Due to the mechanism, a high thrust and vibration can be generated at a wide frequency range. In addition, the manufacturability can be improved due to introduction of a piled coil.

The actuator mainly consists of a mover and a stator. The mover is composed of a yoke, two ring-shaped permanent magnets magnetized in the moving direction, and a shaft made of non-ferromagnetic material. The stator is composed of yokes, two springs, two sets of coils, and supporting parts made of non-ferromagnetic material. The actuator can be easily manufactured because the stator is located outside the mover. As shown in Figure 4, the coil connection can be changed in series or parallel. In the series connection, Coil 1 and Coil 2 are connected with a power supply. In the parallel connection, Coil 1 is connected with a power supply while Coil 2 is connected with a capacitor. Furthermore, electric switching devices are necessary to change the coil connection. Additionally, the number of switching devices increases more than that in the previous actuator because the coil connection is slightly complicated.

**Figure 5:**
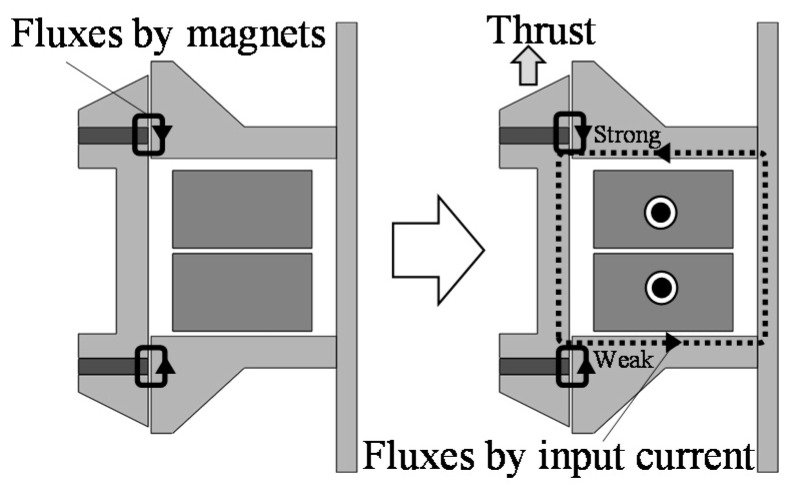
Operational principle in series.

**Figure 6:**
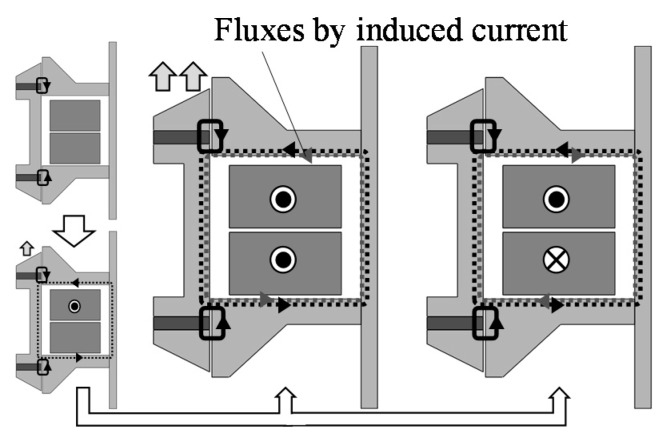
Operational principle in parallel.

The operational principles at each connection are shown in Figure 5 and Figure 6. When the coils are not excited, the magnetic flux distribution around the upper ring magnet is the same as the lower ring magnet, and no thrusts are generated. In the series connection, currents in Coil 1 and Coil 2 produce unbalanced magnetic fluxes, which generate a thrust in the upper direction. In this way, an AC current in the coils generates an AC thrust, and the mover is oscillated [7,12]. In the parallel connection, when the mover is oscillated due to an AC current in Coil 1, an induced voltage is generated, and an induced current flows in Coil 2. In this time, the amplitude and the phase angle between Coil 1 and Coil 2 are different from each other because they depend on the drive frequency. Therefore, Coil 2 can generate thrust, and the mover is oscillated by the thrusts.

In the proposed actuator, a thrust constant is expressed by Equation (1).

(1) $$\frac{F}{I_{\mathrm{input}}}=\frac{A_{1}I_{1}+A_{2}I_{2}}{I_{\mathrm{input}}}$$

where *F* is the thrust, *I*_1_ and *I*_2_ are the currents in Coil 1 and Coil 2, respectively, *I*_input_ is the input current, and *A*_1_ and *A*_2_ are thrust constants of Coil 1 and Coil 2, respectively. In the series connection, the current in Coil 1 and Coil 2 are equal to the input current, and a thrust constant is derived as Equation (2). In the parallel connection, the current in Coil 1 and Coil 2 are equal to the input current and induced current, respectively, and a thrust constant is derived as Equation (3).

(2) $$\frac{F}{I_{\mathrm{input}}}=\frac{A_{1}I_{\mathrm{input}}+A_{2}I_{\mathrm{input}}}{I_{\mathrm{input}}}==A_{1}+A_{2}$$

(3) $$\frac{F}{I_{\mathrm{input}}}=\frac{A_{1}I_{\mathrm{input}}+A_{2}I_{\mathrm{induced}}}{I_{\mathrm{input}}}==A_{1}+A_{2}\frac{I_{\mathrm{induced}}}{I_{\mathrm{input}}}$$

where *I*_induced_ is the induced current. From these equations, a thrust constant is constant in the series connection and is not constant in the parallel connection.

When the actuator is driven at a constant frequency, a thrust constant in the series connection is higher than that in the parallel connection because the phase of the induced current is almost opposite to the input current, and thrusts generated by each current are cancelled in the parallel connection. When the actuator is driven at other frequencies, a thrust constant in the series connection is lower than that in the parallel connection because the phase of the induced current is almost the same as the input current, and thrusts generated by each currents are superimposed in the parallel connection. 

The oscillation of the mover is influenced by the mechanical resonance in the series connection. On the other hand, the oscillation is influenced by the mechanical resonance and induced current in the parallel connection.

From these operations, the frequency characteristics of the thrust and vibration are different and depend on the conditions of the coils. In other words, high thrusts and large vibration can always be generated under lower current by changing the coil connections according to the drive frequency.

## 3. Analysis Method

**Figure 7:**
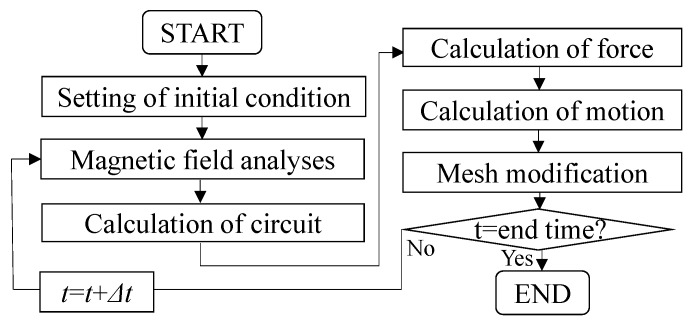
Flowcharts of the analysis method.

In order to verify the operational principle of the proposed actuator, a magnetic field analysis with a motion and circuit equations was conducted as shown in Figure 7 [13,14]. In the magnetic field analysis, the magnetic fluxes are calculated from Maxwell's equation using the 2-D finite element method. Additionally, the electromagnetic force is calculated by the Maxwell's stress method. In the circuit analysis, a current in the series connection is calculated from a circuit equation shown in Equation (4).

(4) $$V=\left(R_{1}+R_{2}\right)I_{\mathrm{input}}+N_{1}{\dot{\psi }}_{1}+N_{2}{\dot{\psi }}_{2}$$

Similarly, the currents in the parallel connection are calculated from the following two circuit equations:

(5) $$V=R_{1}I_{\mathrm{input}}+N_{1}{\dot{\psi }}_{1}$$

(6) $$0=R_{2}I_{\mathrm{induced}}+N_{2}{\dot{\psi }}_{2}+\frac{1}{C}\int I_{\mathrm{induced}}dt$$

Finally, the mover position is calculated from a motion equation shown in Equation (7).

(7) $$M\ddot{x}+D\dot{x}+Kx=F\pm F_{s}$$

where *V* is the applied voltage from the power supply, *N*_1_ and *N*_2_ are the number of turns in Coil 1 and 2, respectively, *R*_1_ and *R*_2_ are the resistance of Coil 1 and Coil 2, respectively, *Ψ*_1_ and *Ψ*_2_ are the interlinkage flux per turn of Coil 1 and Coil 2, respectively, *C* is the capacitance of the capacitor, *M* is the mass of the mover, *D* is the damping coefficient, *K* is the spring constant, and *F*_s_ is the friction force. In the analyses, iron losses are not considered.

## 4. Analyzed Results

A sinusoidal voltage that has an amplitude of 1.2 V and a frequency from 20 to 200 Hz was applied. The analysis conditions are shown in Table 1.

**Figure 8:**
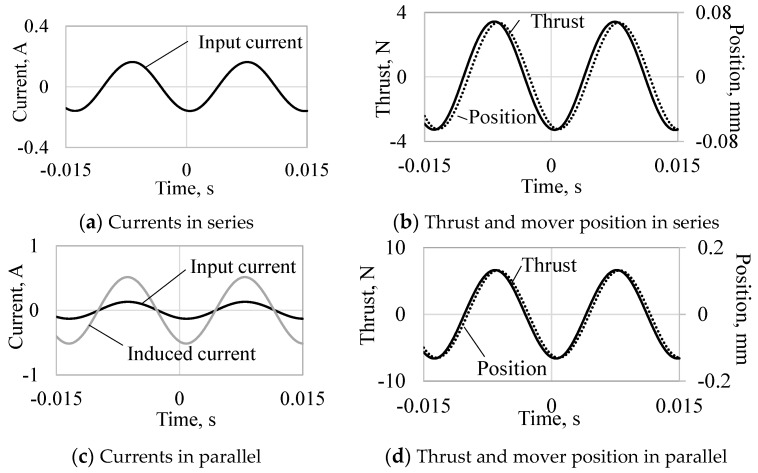
Analyzed results per time variation at 70 Hz.

**Figure 9:**
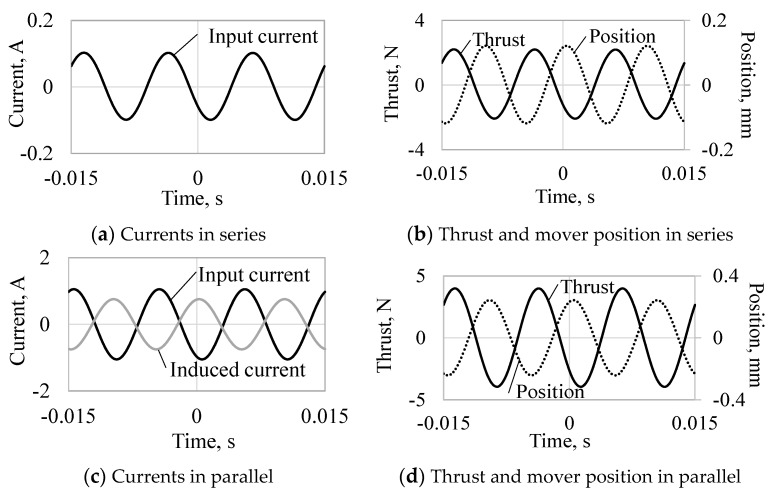
Analyzed results per time variation at 100 Hz.

Figure 8 and Figure 9 show the currents, thrusts, and mover positions at 70 and 100 Hz, respectively. When the actuator is driven at 70 Hz, the thrust constants in the series and parallel connections are 21 N_amp_/A_amp_, and 41 N_amp_/A_amp_, respectively. Furthermore, when the actuator is driven at 100 Hz, the thrust constants in the series and parallel connections are 21 N_amp_/A_amp_ and 3 N_amp_/A_amp_, respectively. The thrust constant in the parallel connection is higher than that in the series connection at 70 Hz because the phase difference of the currents is almost zero and is lower at 100 Hz because the phase difference is opposite, as shown in Figure 8c and Figure 9c. The vibration amplitudes per input current amplitude (vibration constants) in the series and parallel connections are 0.41 mm_amp_/A_amp_ and 0.91 mm_amp_/A_amp_, respectively, at 70 Hz. The vibration constants in the series and parallel connections are 1.18 mm_amp_/A_amp_ and 0.23 mm_amp_/A_amp_, respectively, at 100 Hz. The vibration constant in the series connection is larger at 100 Hz because of the mechanical resonance frequency (96 Hz) depending on the mover weight and the spring stiffness, and that in the parallel connection is larger at 70 Hz due to the higher thrust constant.

**Figure 10:**
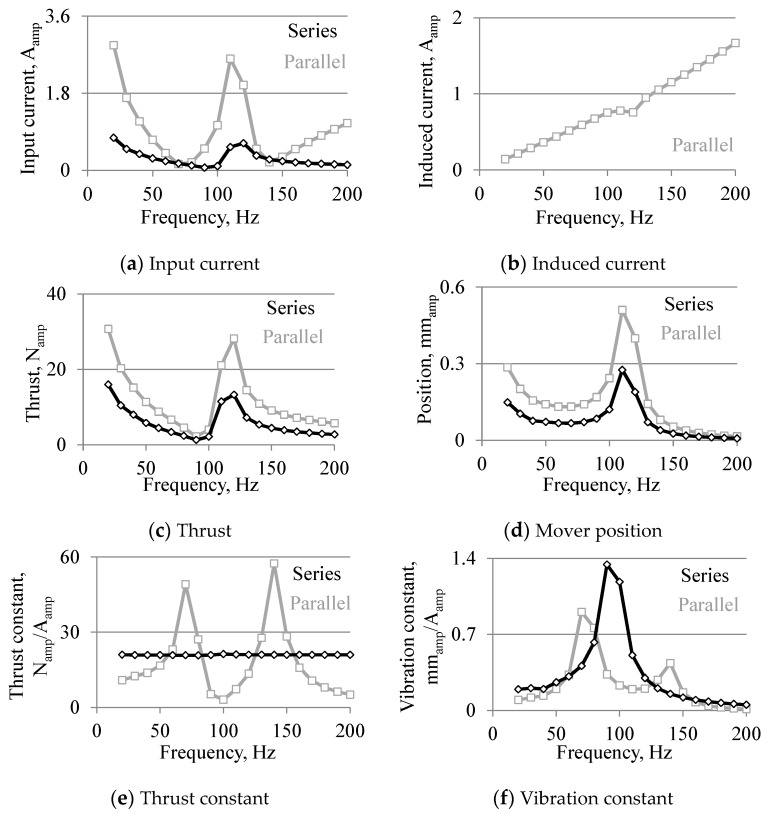
Analyzed results against drive frequency.

Figure 10 shows the analyzed results when the drive frequency is changed. From Figure 10a–d, the input current, thrust, and mover position are influenced by the mechanical resonance, inductances, and capacitor. In addition, the input current, thrust, and mover position in the parallel connection are higher than those in the series connection at almost all drive frequencies because the resistance of the coil that is connected to the power supply is small. Additionally, the input current is extremely small at 70 and 140 Hz in the parallel connection. As shown in Figure 10e, the thrust constant in the series connection is constant with respect to the drive frequency, and that in the parallel has two peaks at 70 and 140 Hz. As shown in Figure 10f, the vibration constant in the series connection has a peak at 100 Hz because of the mechanical resonance, and that in the parallel connection has two peaks at 60 and 140 Hz due to the higher thrust constant. 

Additionally, high thrust and large vibration can be generated by small currents from 60 to 80 Hz, and from 130 to 150 Hz in the parallel connection and at other frequencies in the series connection.

## 5. Conclusions

In this paper, we proposed a new linear oscillatory actuator that can manufacture easily and control characteristics by using two sets of coils. From finite element analysis, it was observed that the thrust constant was constant in the series connection and had two peaks in the parallel connection. Additionally, the vibration constant had a peak in the series connection and had two peaks in the parallel connection. From these results, we verified that the actuator had two different characteristics and could generate high thrust and large vibration using smaller currents at a wide drive frequency range by changing the connection according to the drive frequency.

## Figures and Tables

**Table 1:** Analysis condition.

Coil 1	Resistance (Ω)	0.98
	Number of turns	39
Coil 2	Resistance (Ω)	0.98
	Number of turns	38
Capacitance (mF)		1
Magnetization of magnets (T)		1.3
Mass of Mover (g)		319
Spring constant (N/mm)		106
Damping cofficient (Ns/m)		10
Friction force (N)		0.42
